# Negative Piezoelectric Coefficient in Ferromagnetic 1H-LaBr_2_ Monolayer

**DOI:** 10.1021/acsaelm.1c01214

**Published:** 2022-01-15

**Authors:** Mohammad Noor-A-Alam, Michael Nolan

**Affiliations:** Tyndall National Institute, University College Cork, Lee Maltings, Dyke Parade, T12 R5CP Cork, Ireland

**Keywords:** density functional theory (DFT), piezoelectric monolayer, 2D materials, 2D magnets, negative piezoelectric coefficient

## Abstract

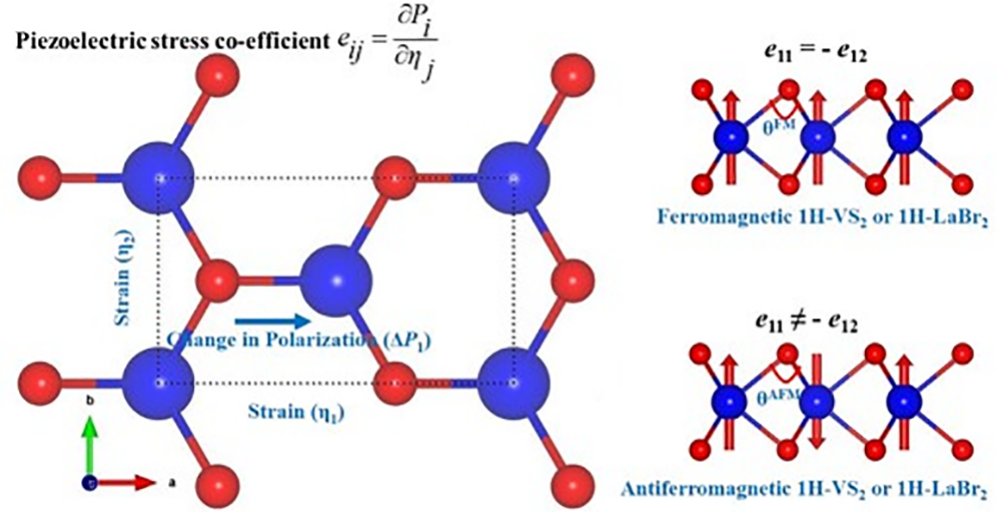

The discovery of two-dimensional (2D) magnetic materials that have excellent piezoelectric response is promising for nanoscale multifunctional piezoelectric or spintronic devices. Piezoelectricity requires a noncentrosymmetric structure with an electronic band gap, whereas magnetism demands broken time-reversal symmetry. Most of the well-known 2D piezoelectrics, e.g., 1H-MoS_2_ monolayer, are not magnetic. Being intrinsically magnetic, semiconducting 1H-LaBr_2_ and 1H-VS_2_ monolayers can combine magnetism and piezoelectricity. We compare piezoelectric properties of 1H-MoS_2_, 1H-VS_2_, and 1H-LaBr_2_ using density functional theory. The ferromagnetic 1H-LaBr_2_ and 1H-VS_2_ monolayers display larger piezoelectric strain coefficients, namely, *d*_11_ = −4.527 pm/V for 1H-LaBr_2_ and *d*_11_ = 4.104 pm/V for 1H-VS_2_, compared to 1H-MoS_2_ (*d*_11_ = 3.706 pm/V). 1H-MoS_2_ has a larger piezoelectric stress coefficient (*e*_11_ = 370.675 pC/m) than 1H-LaBr_2_ (*e*_11_ = −94.175 pC/m) and 1H-VS_2_ (*e*_11_ = 298.100 pC/m). The large *d*_11_ for 1H-LaBr_2_ originates from the low elastic constants, *C*_11_ = 30.338 N/m and *C*_12_ = 9.534 N/m. The sign of the piezoelectric coefficients for 1H-LaBr_2_ is negative, and this arises from the negative ionic contribution of *e*_11_, which dominates in 1H-LaBr_2_, whereas the electronic part of *e*_11_ dominates in 1H-MoS_2_ and 1H-VS_2_. We explain the origin of this large ionic contribution of *e*_11_ for 1H-LaBr_2_ through Born effective charges (*Z*_11_) and the sensitivity of the atomic positions to the strain (d*u*/dη). We observe a sign reversal in the *Z*_11_ values of Mo and S compared to the nominal oxidation states, which makes both the electronic and ionic parts of *e*_11_ positive and results in the high value of *e*_11_. We also show that a change in magnetic order can enhance (reduce) the piezoresponse of 1H-LaBr_2_ (1H-VS_2_).

## Introduction

Piezoelectric materials are used in a wide range of important devices such as microphones, medical imaging, and sensors.^1,2^ Recently it has been demonstrated that the piezopotential originating from piezoelectricity can be used as a gate voltage to control the electronic band gap of a piezoelectric semiconductor, opening a new field of research named “piezotronics”.^1,2^ In this regard, 2D semiconductors are promising materials as they can sustain the large deformations present in piezoelectric applications.^1,2^ Moreover, these 2D materials show unique optical properties, for example, valleytronics.^3,4^ Hence, 2D materials can be ideal for piezophotonics where charges stemming from the piezoelectric effect can couple with light to significantly modulate the charge-carrier generation, separation, transport, and/or recombination in semiconducting nanostructures, promising better LEDs, photodetectors, and solar cells.^1,2^

Piezoelectricity and valleytronics require broken inversion symmetry and a band gap. Promisingly, there already exists a wide range of noncentrosymmetric and intrinsically piezoelectric 2D materials.^3,4^ On the other hand, there are only a few 2D semiconductors/insulators to date in which both time-reversal and inversion symmetry are broken. These noncentrosymmetric magnetic 2D materials, e.g., vanadium dichalcogenide monolayers,^5^ exhibit spontaneous valley polarization, which can be controlled by a magnetic field.^3^ Very recently, the coexistence of magnetism and piezoelectricity has also been predicted in vanadium dichalcogenide monolayers.^6^ However, how the magnetic ordering impacts on their piezoelectricity remains unexplored. This understanding will allow us to couple magnetism and piezoelectricity for realizing multifunctional piezoelectric devices.

A piezoelectric stress coefficient (*e*_*ij*_), defined as , where ∂*P*_*i*_ is the induced polarization along the *i*-direction in response to strain ∂*η*_*j*_ along the *j*-direction, can be split into two contributions: the ionic part, *e*_*ij*_^ion^, where ions are allowed to move under an applied strain, and the electronic part (also known as the clamped-ion part) *e*_*ij*_^elc^, where ions are clamped under applied strain. In many bulk materials, including wurtzite nitrides,^7,8^*e*_*ij*_^elc^ is negative but is dominated by positive *e*_*ij*_^ion^, thus resulting in a positive value of *e*_*ij*_. Generally, a positive longitudinal piezoelectric coefficient is expected as a tensile strain is expected to increase the induced electric polarization. However, very recently an anomalous negative piezoelectric coefficient has been observed in the layered ferroelectric CuInP_2_S_6_,^7^ which is explained in terms of its large negative *e*_*ij*_^elc^ that is not overcome by positive *e*_*ij*_^ion^. Also, negative piezoelectric coefficients—due to their large negative *e*_*ij*_^elc^’s—have been observed in several hexagonal ABC ferroelectrics.^9^ A negative longitudinal piezoelectric coefficient would mean that the material contracts along the direction of an applied electric field rather than expands. This can enable novel nanoscale electromechanical devices, e.g., piezoelectric actuators.

This raises an interesting question: can a negative total *e*_*ij*_ be obtained due to large negative *e*_*ij*_^ion^ instead of *e*_*ij*_^elc^? To answer this question, we investigate three intrinsically piezoelectric monolayers, 1H-MoS_2_, 1H-VS_2_, and 1H-LaBr_2_, and we discover 1H-LaBr_2_ as a new 2D piezoelectric monolayer that has a negative piezoelectric coefficient originating from a large negative *e*_*ij*_^ion^. Being a magnetic, semiconducting electride, 1H-LaBr_2_ is a unique monolayer, although it has not been achieved experimentally yet; however, it is predicted to be feasible via chemical exfoliation from its layered bulk structure.^10^ It combines peculiar features; for example, its electron density shows neither complete localization at an atomic site nor metal-like delocalization, but rather it occupies the center of the hexagon from which originate localized magnetic moments.^11,12^ Very recently, it has been predicted that this magnetism can be utilized for valley polarization.^10^ However, its piezoelectric properties have not been investigated to date.

Recently a number of 2D materials in the 1H structure (*D*_3*h*_ symmetry) have been predicted to show large piezoelectric co-coefficients.^13−16^ These 2D materials still remain at the stage of fundamental research; understanding the origin of piezoelectricity can promote the discovery of more 2D piezoelectrics. Encouragingly, piezoelectricity has also been experimentally confirmed in the 1H-MoS_2_ monolayer,^17^ and the value *e*_11_ (2.9 × 10^–10^ C/m) is in good agreement with first-principles calculations of *e*_11_ = 3.64 × 10^–10^ C/m.^18^ Recently, the coexistence of magnetism and piezoelectricity has also been predicted in the 1H-VS_2_ monolayer,^6^ although the coupling between magnetic order and piezoelectricity was not discussed. Note that research on these 1H structured 2D piezoelectrics is mainly devoted to finding large piezoelectric coefficients, overlooking their sign as they generally show positive in-plane piezoelectric coefficients.^6,13−16,18^

However, the origin of the piezoelectric co-coefficients in both magnitude and sign still remains unclear. Questions include the following: Why is the *e*_11_ of the 1H-MoS_2_ monolayer larger than that of the 1H-VS_2_ monolayer? Why is the sign of the ionic part of *e*_11_ positive in the 1H-MoS_2_ monolayer but negative in the 1H-VS_2_ monolayer? In this paper, we show that the answers to these questions have their origin in the Born effective charges (BECs), the sensitivity of the atomic positions in response to a strain , and the bond strength. We also demonstrate that the 1H-LaBr_2_ monolayer^10−12^ can be a magnetic, piezoelectric material. Moreover, we show that antiferromagnetic ordering makes the isotropic piezoelectricity of the ferromagnetic 1H-LaBr_2_ monolayer anisotropic (i.e., *e*_11_ ≠ −*e*_12_).

## Computational Details

**Figure 1:**
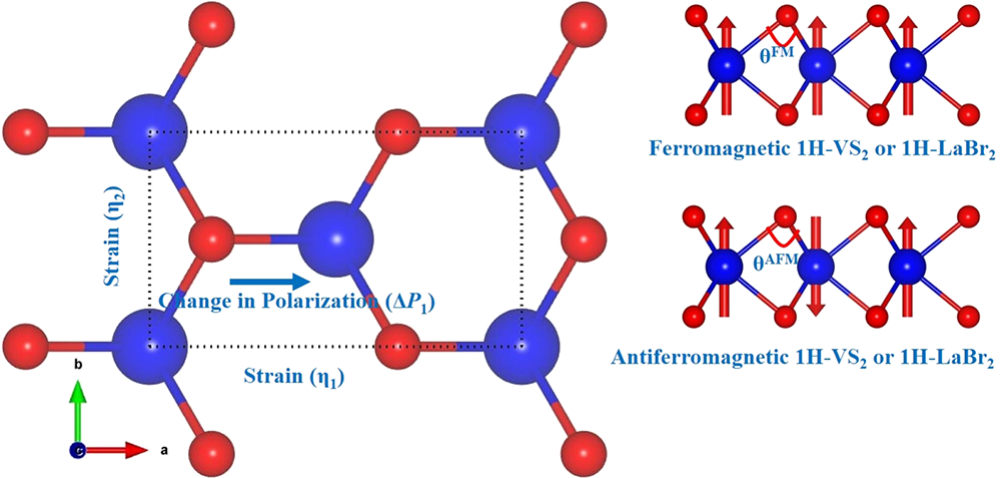
Rectangular unit cell. Blue and red balls represent Mo/V/La and S/O, respectively. The red arrows represent up and down collinear spin states of an atom.

Our first-principles calculations are performed in the framework of spin-polarized density functional theory using projector augmented wave (PAW) potentials^19^ to describe the core electrons and the generalized gradient approximation (GGA) of Perdew, Burke, and Ernzernhof (PBE)^20^ for exchange and correlation as implemented in the Vienna Ab initio Simulation Package (VASP) based on a plane-wave basis set.^21^ The valence electron configurations for La, V, Mo, S, and Br are 4p^6^5s^2^6d^1^ (nine electrons), 3p^6^3d^4^4s^1^ (11 electrons), 4p^6^4d^5^5s^1^ (12 electrons), 3s^2^3p^4^ (six electrons), and 4s^2^4p^5^ (seven electrons), respectively. A cutoff energy of 500 eV for the plane-wave expansion is used in all calculations, and all structures are fully relaxed until the Hellmann–Feynman forces on all the atoms are less than 10^–3^ eV/Å. An effective onsite Coulomb interaction parameter (*U*_eff_) of 6.5 eV is used for the La f-electrons.^11^ The lattice parameters and internal coordinates of the 2D structures are fully relaxed to achieve the lowest energy configuration using the conjugate gradient algorithm. To prevent the interaction between the periodic images in the calculations, a vacuum layer with a thickness of approximately 25 Å is added along the *z*-direction (perpendicular to the monolayer) in the supercell. Note that a rectangular cell (see Figure 1) is used instead of a primitive hexagonal one for applying strain along the desired direction. This is a commonly used approach.^6,18^ Geometry optimization is carried out employing the conjugated gradient technique, and the convergence for the total energy is set as 10^–7^ eV. The Brillouin zone integration is sampled using a regular 6 × 8 × 1 Monkhorst–Pack *k*-point grid, for geometry optimizations, while a denser grid of 12 × 16 × 1 is used for density functional perturbation theory (DFPT) calculations. The elastic stiffness coefficients (*C*_*ij*_) are obtained with a finite difference method as implemented in the VASP code. DFPT as implemented in the VASP code is used to calculate Born effective charges (*Z*_*ij*_) and ionic and electronic parts of piezoelectric (*e*_*ij*_) tensors.

## Results and Discussion

Table 1 shows the lattice parameters of the monolayers. We use a rectangular unit cell, and lattice parameter *a* should be equal to  for an ideal 1H structure. Our calculated lattice parameters are in good agreement with previously reported values.^6,10−12,18^ 1H-LaBr_2_ has significantly larger lattice parameters compared to these of the other two monolayers—mainly because the ionic radius of La (Br) is larger than that of Mo/V (S) according to the Database of Ionic Radii (http://abulafia.mt.ic.ac.uk/shannon/ptable.php). However, we notice that strip antiferromagnetic (AFM) ordered structures shown in Figure 1 deviate from the ideal relationship, shrinking along the zigzag, or *b*-axis, direction and expanding along the armchair, or *a*-axis, direction. We quantify this deviation as
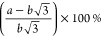
This is about 1.22% (0.72%) for the AFM 1H-LaBr_2_ (1H-VS_2_) monolayer. This deviation is also reflected in the change in angles θ^FM^ and θ^AFM^, which are defined in Figure 1.

**Table 1:** Structural Information of the Monolayers: Optimized Lattice Parameters *a* and *b*^a^ and the Angle ∠Mo/V/La–S/Br–Mo/V/La^b^

	*a* (Å)	*b* (Å)	θ^FM^ (deg)	θ^AFM^ (deg)
1H-MoS_2_	5.522	3.188	–	–
1H-VS_2_(FM)	5.504	3.178	84.372	–
1H-VS_2_(AFM)	5.502	3.154	84.177	84.518
1H-LaBr_2_(FM)	7.298	4.214	84.189	–
1H-LaBr_2_(AFM)	7.344	4.189	83.624	84.556

a See the rectangular cell in Figure 1.

b θ^FM^ (θ^AFM^) is the angle ∠V↑/La↑–S/Br–V↑/La↑ (∠V↑/La↑–S/Br–V↓/La↓), where ↑ and ↓ arrows represent up and down spin polarizations. The angle ∠Mo–S–Mo is 82.537°.

In agreement with previous reports,^6,10−12^ we find that ferromagnetic (FM) ordering is the ground state for both 1H-LaBr_2_ and 1H-VS_2_ monolayers, lying 51.520 and 88.545 meV lower in energy compared to the strip AFM state. However, the magnetic order of these monolayers has not been clearly identified in experiments to date. Although the VS_2_ monolayer has not yet been synthesized, ferromagnetism has been recently found in its ultrathin films.^22,23^

The 1H-LaBr_2_(FM), 1H-VS_2_(FM), and 1H-MoS_2_ monolayers belong to the nonmagnetic space group *P*6*m*2 (157), that is, considering their structures but without their magnetic order. Unlike the corresponding bulk materials, this structure has no inversion symmetry and is intrinsically piezoelectric. We calculated their piezoelectric stress coefficients, which are shown in Table 2. The piezoelectric coefficients that involve strain along the *z*-direction are ill-defined for the monolayers. Our 1H monolayers have only one independent piezoelectric coefficient, *e*_11_ (*e*_11_ = −*e*_12_), due to 6̅*m*2 point group symmetry. Table 2 shows that 1H-MoS_2_ has a quite large *e*_11_ value compared to those of the other two monolayers. Interestingly, FM 1H-LaBr_2_ shows a negative *e*_11_ which is also quite low compared to those of the other materials. To understand the origin of piezoelectric constant, *e*_11_ and *e*_12_ can be decomposed into two parts:^8,24^
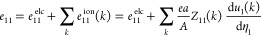
1
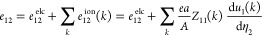
2

**Table 2:** Electronic (*e*_11_^elc^) and Ionic (*e*_11_^ion^) Parts of the Total Piezoelectric Stress Constant *e*_11_^a^ and Born Effective Charge *Z*_11_^b^^,^^c^

	*e*_11_^elc^	*e*_11_^ion^	*e*_11_	*Z*_11_(M)	*Z*_11_(X)	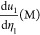	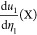
1H-MoS_2_	315.000	56.050	371.050	–1.006	0.503	–0.037	0.018
1H-VS_2_(FM)^d^	379.025	–80.925	298.100	1.359	–0.680	–0.038	0.021
1H-LaBr_2_(FM)^d^	111.175	–205.350	–94.175	2.540	–1.269	–0.069	0.035

a In 2D piezoelectric unit, pC/m.

b M = Mo, V, and La and X = S and Br in |*e*|, where *e* is the charge of an electron.

c Here,  represents the change of the position of the atoms along the *a*-direction under a strain along the *a*-direction (η_1_).

d Both 1H-VS_2_ and 1H-LaBr_2_ monolayers are in the ferromagnetic (FM) state.

The clamped-ion term (*e*_11_^elc^ or *e*_12_^elc^) arises from the contributions of electrons when the ions are frozen at their zero-strain equilibrium internal atomic coordinates (*u*), and the internal-strain (*e*_11_^ion^) term arises from the contribution from internal microscopic atomic displacements in response to a macroscopic strain. In our case, the strain (η_1_) is applied in the *x*-direction (see Figure 1). Here, *k* runs over all the atoms in the unit cell, *a* is the in-plane lattice constant, *e* is the electron charge, and *A* is the area as the 2D unit is used. The Born effective charge (*Z*_11_(*k*)) of the *k*th atom is calculated by the DFPT approach. The response of the *k*th atom’s internal coordinate along the *x*-direction (*u*_1_(*k*)) in response to a macroscopic strain (η_1_) is measured by . Table 2 shows that both *e*_11_^elc^ and *e*_11_^ion^ have the same sign—positive—for 1H-MoS_2_, unlike the other two monolayers. This results in a large total *e*_11_ for 1H-MoS_2_. Note that other 1H-MX_2_ (M = Mo, W, Cr and X = S, Se, Te) monolayers also exhibit positive *e*_11_^elc^ and *e*_11_^ion^ (see Table S1).^18^ However, due to the opposite signs of *e*_11_^elc^ and *e*_11_^ion^ in 1H-VS_2_(FM), its total *e*_11_ is smaller than the *e*_11_ for 1H-MoS_2_, even though it has a larger value of *e*_11_^elc^ (see Table 2). Interestingly, 1H-LaBr_2_(FM) shows a negative *e*_11_^ion^, which is significantly larger than its *e*_11_^elc^, thus resulting in a negative total *e*_11_. This is different from the recently discovered negative piezoelectric coefficient in layered ferroelectrics and wurtzite, where the negative sign comes from *e*_11_^elc^.^7^ Here it is important to highlight that other 2D piezoelectrics, e.g., the well-known hexagonal boron nitride (h-BN) monolayer^18^ and the 1H-VSe_2_ monolayer,^6^ have negative *e*_11_^ion^ values but the *e*_11_^elc^ part dominates, resulting in positive *e*_11_. Interestingly, we find that h-AlN and h-ZnO monolayers also exhibit negative *e*_11_ values due to large negative *e*_11_^ion^ values (see Table S1). We hope that our finding will inspire experimental studies of negative in-plane piezoresponse (*e*_11_) in 2D materials. Deepening our understanding about the 2D piezoelectrics, this would enable discovery of 2D materials for novel electromechanical applications.

Now to understand the origin of negative/positive *e*_11_^ion^, we expressed *e*_11_^ion^ in terms of *Z*_11_ and  (see eq 1). Providing microscopic insight into the piezoelectric coefficients, BEC is a dynamical charge that is directly related to the change of electric polarization or dipole moment (for molecules) in response to an atomic displacement.^25^*Z*_11_(*k*) is proportional to , where *∂P*_1_ is the change of the dipole moment in the *x*-direction induced by a small displacement of atom *k* in the same direction (*∂τ*_1_(*k*)).^25^ The negative slope  will result in a negative BEC, which is the case for 1H-MoS_2_. This proportionality (i.e., the slope) is the origin of BECs and has the dimensionality of an electric charge. This charge is a well-defined and experimentally measurable quantity—owing to the fact that the BECs are related to LO–TO splitting, which is the frequency difference between the longitudinal (LO) and transverse (TO) optical phonon modes.^25^ From Table 2, it is clear that the positive *e*_11_^ion^ in 1H-MoS_2_ is due to unusual BECs of Mo and S as we see a negative (positive) sign for cation Mo (anion S) in the BECs. Such counterintuitive BECs—Mo (S) shows a negative (positive) dynamical charge, opposite to its static positive charge—are also reported for bulk 2H-MoS_2_.^26^ Interestingly, we notice that other good 2D piezoelectric transition metal (Mo, W, and Cr) dichalcogenide (S, Se, and Te) monolayers also exhibit counterintuitive BECs (see Table S1), making their *e*_11_^ion^ values positive; thus, both *e*_11_^ion^ and *e*_11_^elc^ positively contribute to the total *e*_11_. We also observe the previously reported trend that the larger the chalcogen is, the larger the *e*_11_. This is mainly because the larger the chalcogen is, the larger the *e*_11_^ion^ due to larger *Z*_11_ and  (see Table S1). Compared to 1H-LaBr_2_(FM), the magnitude of *e*_11_^ion^ in 1H-MoS_2_ and 1H-VS_2_(FM) is smaller because of smaller BECs and . We find that the large negative *e*_11_^ion^ in 1H-LaBr_2_(FM) originates from its large *Z*_11_ and ; both terms are almost 2 times larger than those of 1H-MoS_2_ or 1H-VS_2_(FM). The large  of 1H-LaBr_2_(FM) can be due to its weaker La–Br bond (see Table 3) indicated by the integrated crystal orbital Hamilton population (ICOHP). In addition, compared to the other two monolayers, its larger lattice parameters (see Table 1) can promote larger displacement of atoms in response to strain as atoms have more space to move. We propose that BECs and lattice parameters—rather than static charges like Bader charge—can be ideal descriptors for searching for improved 2D piezoelectrics as they are directly related to the *e*_*ij*_ and can be routinely computed, allowing for automated and high-throughput screening. Our results also explain the previous observation that there is no significant correlation of *d*_11_ with electronegativity or Bader charges, whereas *d*_11_ shows a strong correlation with polarizabilities of anions and cations.^13^ Note that BECs can also be considered as a manifestation of local polarizabilities of atoms.^25^

**Table 3:** Elastic Constants (*C*_11_ and *C*_12_), ICOHP of a Bond between Cation (Mo/V/La) and Anion (S/Br), Poisson’s ratio ν (=*C*_12_/*C*_11_), and Piezoelectric Strain Coefficient *d*_11_

	*C*_11_ (N/m)	*C*_12_ (N/m)	ICOHP (eV/bond)	ν	*d*_11_ (pm/V)
1H-MoS_2_	133.214	33.105	–3.113	0.249	3.706
1H-VS_2_(FM)	101.421	28.785	–2.510	0.284	4.104
1H-LaBr_2_(FM)	30.338	9.534	–1.919	0.314	–4.527

Now we calculate the piezoelectric stress constants (*d*_*ij*_) using *e*_*ij*_ and elastic constants (*C*_*ij*_) (see Table 3). First, the mechanical/elastic stability of the ferromagnetic (FM) 1H-LaBr_2_ monolayer is checked according to the criteria for a 2D hexagonal crystal structure:^27^*C*_11_ > *C*_12_ and *C*_66_ > 0. Considering the two independent elastic constants (only two independent elastic constants due to space group *P*6*m*2 and two-dimensionality) that we obtain, namely, *C*_11_ = 30.34 N/m and *C*_12_ = 9.53 N/m (notice that *C*_66_ = (*C*_11_ – *C*_12_)/2), it can be concluded that the monolayer is mechanically stable. The dynamic stability of 1H-LaBr_2_ in terms of phonon modes has already predicted.^10^ Our calculated elastic coefficients for the monolayers are in good agreement with the previously reported values.^6,10,18^ Compared to the 1H-MoS_2_ and 1H-VS_2_(FM) monolayers, its lower *C*_11_ and *C*_12_ values but larger ν indicate that the 1H-LaBr_2_(FM) monolayer is much softer. This is also expected because of its larger lattice parameters. This softening of elastic coefficients can also be understood from bond strength analysis. For that, we use the ICOHP approach,^28^ which allows us to quantify the strength of the covalency of a bond. The more negative ICOHP, the stronger the covalent bonding. Here we emphasize that ICOHP is a reasonable qualitative estimation of the bond strength but it is not the bond enthalpy. We see in Table 3 that the 1H-LaBr_2_(FM) monolayer has a significantly weaker La–Br bond, with an ICOHP of −1.919 and a La–Br bond length of 3.14 Å, compared to those of Mo–S, with an ICOHP of −3.113 and a Mo–S distance of 2.417 Å, or V–S, with an ICOHP of −2.51 and a V–S distance of 2.366 Å. Table 3 shows that *d*_11_ (again the only independent coefficient due to symmetry and dimensionality; ) of 1H-LaBr_2_(FM) is about 22% larger than that of the well-known 2D piezoelectric 1H-MoS_2_ because the former has quite low elastic constants. The origin of the negative sign in *d*_11_ of 1H-LaBr_2_(FM) is in its negative *e*_11_, which is discussed above. As also previously reported,^18^ despite being ultrathin, the piezocoefficients of these 2D piezoelectrics are comparable with those of well-known bulk piezoelectrics, e.g., α-quartz (*d*_11_ = 2.3 pm/V)^29^ and wurtzite nitrides such as AlN (*d*_33_ = 5.1 pm/V)^30^ and GaN (*d*_33_ = 3.1 pm/V).^30^

Now we discuss how the magnetic ordering can affect the piezoelectric response. We consider simple strip-type antiferromagnetic (AFM) order (see Figure 1). The calculated values of *e*_11_ and *e*_12_ are shown in Table 4. Interestingly, we find that *e*_11_ is not equal to −*e*_12_ for AFM, whereas *e*_11_ = −*e*_12_ for FM. Moreover, *e*_11_ in AFM is quite different from *e*_11_ in FM (see Table 4). For example, *e*_11_ of AFM 1H-LaBr_2_ is almost double compared to that of FM; however, *e*_11_ is still negative. To understand the origin of *e*_11_ ≠ – *e*_12_ for AFM, we consider two cases: (i) the structures (lattice parameters *a* and *b* and atomic positions) are relaxed and (ii) AFM order is used, keeping the lattice parameters *a* and *b* and atomic positions fixed in their FM structures, which are represented by asterisks in Table 4. We see that it is the change in magnetic order that intrinsically causes *e*_11_ ≠ – *e*_12_ for AFM, not the structural changes associated with this magnetic order change, although the structural relaxation changes the values, too. Both *e*^elc^ and *e*^ion^ change (both *e*_11_^ion^ ≠ −*e*_12_^ion^ and *e*_11_^elc^ ≠ −*e*_12_^elc^ for AFM) in response to the change in magnetic order. Table 4 also shows how the BECs and  change, resulting in changes to *e*^ion^. We notice that the magnitude of *Z*_11_ for both La (V) and Br (S) in AFM order has increased (decreased), promoting enhancement in total *e*_11_ or *e*_12_.

**Table 4:** Electronic (*e*_11_^elc^ and *e*_12_^elc^) and Ionic (*e*_11_^ion^ and *e*_12_^ion^) Parts of the Total Piezoelectric Stress Constants *e*_11_ and *e*_12_ of Antiferromagnetic 1H-VS_2_ and 1H-LaBr_2_ Monolayers^a^ and Born Effective Charge *Z*_11_^b^^,^^c^

	*e*_11_^elc^	*e*_11_^ion^	*e*_11_	*Z*_11_(M)	*Z*_11_(X)	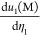	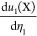	*e*_12_^elc^	*e*_12_^ion^	*e*_12_	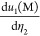	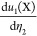
1H-VS_2_	221.900	–38.375	183.525	0.662	–0.210	–0.031	0.015	–284.150	12.175	–271.975	0.025	–0.013
1H-VS_2_*^d^	218.075	–14.525	203.550	0.657	–0.203	–0.033	0.016	–280.825	40.200	–240.625	0.026	–0.013
1H-LaBr_2_	43.650	–269.025	–225.375	2.765	–1.381	–0.079	0.040	–96.225	199.300	103.075	0.066	–0.033
1H-LaBr_2_*^d^	57.275	–281.000	–223.725	2.744	–1.370	–0.083	0.041	–100.075	197.700	97.625	0.068	–0.034

a In 2D piezoelectric unit, pC/m.

b M = Mo, V, and La and X = S and Br in |*e*|, where *e* is the charge of an electron.

c Here,  represents the change of the position of the atoms along the *a*-direction under a strain along the *b*-direction (η_2_).

d 1H-VS_2_* and 1H-LaBr_2_* represent antiferromagnetic 1H-VS_2_ and 1H-LaBr_2_ monolayers in their ferromagnetic structures (i.e., just the magnetic order is changed, no structural relaxation).

We also calculate the *d*_*ij*_ coefficients (see Table 5) using the relation
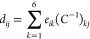
We notice that *C*_11_ is not equal to *C*_22_ for AFM structures. As *e*_11_ ≠ −*e*_12_ and *C*_11_ ≠ −*C*_22_ for AFM structures, *d*_11_ ≠ −*d*_12_ for AFM as shown in Table 5 is expected. We find that the *d*_11_ (also *e*_11_) of AFM 1H-LaBr_2_ is about 2 times larger than that of its FM. We believe that such a change in piezoresponse induced by magnetic order can also be observed in other magnetic 2D piezoelectrics. In experiments, the magnetic direction (noncollinear magnetic order) can play a vital role, which is beyond the scope of this paper. Note that changing the magnetic order in a controlled way experimentally might be a challenge—especially for the change from FM to AFM. However, a transition from the AFM state to the FM state can be achieved by applying an external magnetic field to the AFM ordered samples.

**Table 5:** Elastic Constants (*C*_11_, *C*_22_, and *C*_12_) and Piezoelectric Strain Coefficients (*d*_11_ and *d*_12_)

	*C*_11_ (N/m)	*C*_22_ (N/m)	*C*_12_ (N/m)	*d*_11_ (pm/V)	*d*_12_ (pm/V)
1H-VS_2_(AFM)	94.586	105.378	32.867	2.832	–3.359
1H-LaBr_2_(AFM)	28.005	31.198	8.981	–10.343	6.137

## Conclusion

We show that the 1H-LaBr_2_ monolayer exhibits an unusual in-plane negative piezoelectric coefficient, unlike many other 1H structured 2D piezoelectrics.^13−16^ This would mean that the monolayer contracts along the *x*-direction (armchair direction) rather than expands, when an electric field is applied in the *x*-direction. Here the origin of the negative piezoelectric coefficient is because of a large negative *e*_11_^ion^ that cannot be compensated by *e*_11_^elc^; this is different from hitherto observed negative piezocoefficients in some bulk materials due to large *e*_*ij*_^elc^ values.^7,9^ The 1H-LaBr_2_ monolayer is a promising 2D piezoelectric, having a large piezoelectric *d*_11_ (−4.527 pm/V) coefficient, which is comparable to those of well-known 2D piezoelectric 1H-MoS_2_ and 1H-VS_2_ monolayers and is larger than that of bulk wurtzite GaN (*d*_33_ ∼ 3.1 pm/V). We also explain the origin—both sign and magnitude—of the piezoelectric coefficients of three monolayers (1H-LaBr_2_, 1H-MoS_2_, and 1H-VS_2_) in terms of their dynamical charges (BECs) and atomic sensitivity (d*u*/dη) to an applied strain. Being directly linked with *e*_*ij*_, we propose that BECs, rather than a static charge like the Bader charge, which relate to atom polarizability, can be good descriptors for searching new 2D piezoelectrics, also providing insight into the underlying mechanism. The calculation of BECs can be automated to allow for high-throughput screening. Additionally, we show that a change in magnetic order can have an effect on their piezoresponse quite significantly, which can be a unique way for coupling magnetism and electromechanical properties in 2D magnets.

## Supporting Information Available

Electronic (*e*_11_^elc^) and ionic (*e*_11_^ion^) parts of total piezoelectric stress constant *e*_11_; Born effective charge *Z*_11_ and d*u*_1_/dη_1_ of 1H-MoS_2_, 1H-MoSe_2_, 1H-MoTe_2_, 1H-WS_2_, 1H-WSe_2_, 1H-WTe_2_, 1H-CrS_2_, 1H-CrSe_2_, 1H-CrTe_2_, h-BN, h-AlN, and h-ZnO (PDF)
