# Supplementary material for: Negative Piezoelectric Coefficient in Ferromagnetic 1H-LaBr2 Monolayer
Source: ACS Appl Electron Mater. 2022 Jan 15;4(2):850–5. doi: 10.1021/acsaelm.1c01214 (PMC8867721; doi:10.1021/acsaelm.1c01214)
Supplement: Supplementary file 1 — el1c01214_si_001.pdf [file el1c01214_si_001.pdf]

Supporting Information

Negative Piezoelectric Coefficient in Ferromagnetic 1H-$LaBr_2$ Monolayer

Mohammad Noor-A-Alam* and Michael Nolan*

*Tyndall National Institute, Lee Maltings, Dyke Parade, University College Cork, Cork, T12 R5CP, Ireland, *Corresponding author: Mohammad Noor-A-Alam (mda.alam@tyndall.ie) and Michael Nolan (michael.nolan@tyndall.ie)*

E-mail:

Table S1: The electronic (e_{11}^{elc}) and ionic (e_{11}^{ion}) part of the total piezoelectric stress constant e_{11} in 2D piezoelectric unit pC/m, born effective charge Z_{11} of M= Mo, W, Cr, B, Al, and Zn and X= S, Se, Te, N, and O in $|e|$ unit, where e is the charge of an electron. $\frac{du_1}{d\eta_1}$ represents the change of the position of the atoms along a-direction under a strain along a-direction (η_1).

	e_{11}^{elc}	e_{11}^{ion}	e_{11}	Z_{11}(M)	Z_{11}(X)	$\frac{du_1}{d\eta_1}$(M)	$\frac{du_1}{d\eta_1}$(X)
1H-MoS_2	315.000	56.050	371.050	-1.006	0.503	-0.037	0.018
1H-$MoSe_2$	277.325	112.100	389.425	-1.794	0.893	-0.043	0.022
1H-$MoTe_2$	252.025	223.700	475.725	-3.242	1.616	-0.052	0.026
1H-WS_2	231.700	28.100	259.800	-0.495	0.248	-0.038	0.019
1H-WSe_2	194.175	76.100	270.275	-1.201	0.597	-0.044	0.022
1H-WTe_2	156.250	183.675	339.925	-2.581	1.287	-0.053	0.026
1H-CrS_2	419.050	132.750	551.800	-2.434	1.211	-0.035	0.017
1H-$CrSe_2$	401.350	182.900	584.250	-3.072	1.514	-0.041	0.021
1H-$CrTe_2$	408.600	288.825	697.425	-4.319	2.054	-0.050	0.025
h-BN	370.950	-232.350	138.600	2.709	-2.709	-0.034	0.034
h-AlN	152.000	-379.775	-227.775	2.713	-2.712	-0.068	0.068
h-ZnO	188.675	-445.825	-257.150	2.485	-2.484	-0.094	0.094
